# Two-year outcomes of surgeon-tailored trans obturator tape for female stress urinary incontinence: a randomized, comparative, trial with traditional trans obturator tape

**DOI:** 10.1186/s12894-021-00922-4

**Published:** 2021-11-13

**Authors:** Salah E. Shebl

**Affiliations:** Urology Department, Faculty of Medicine for Girls, Al-Azhar University, Cairo, Egypt

**Keywords:** Stress urinary incontinence, Trans obturator tape, Long-term

## Abstract

**Background:**

Previously, we presented the short-term outcomes of surgeon-tailored mesh in patients with SUI undergoing TOT. In this report, we aim to highlight the two-year outcomes of surgeon tailored mesh in terms of subjective and objective cure rates, as well as late complications.

**Methods:**

We performed a randomized, open-label comparative trial that recruited women with SUI who were scheduled to undergo TOT. Eligible patients were randomly allocated in a 1:1 ratio to receive traditional TOT mesh or surgeon-tailored polyethylene mesh. All patients were followed up for two years.

**Results:**

At the end of the follow-up, there were 13 women in the traditional TOT mesh group and 14 patients in the surgeon-tailored polyethylene mesh group. Concerning the primary outcome of the present study, the cure rate was 100% in the surgeon-tailored polyethylene mesh (n = 14) and 92.9% in the traditional TOT mesh group (*p* = 0.39). One woman reported improved symptoms in the traditional TOT mesh group. There were no reported failures in both groups. Concerning safety, the incidence of de novo urgency was 0% in the surgeon-tailored polyethylene mesh group, compared to 7.1% in the traditional TOT mesh group (*p* = 0.34). None of the women in both groups reported mesh erosions, dyspareunia, or need for reoperation.

**Conclusion:**

Surgeon-tailored mesh for patients undergoing TOT is a cost-effective technique, which has comparable long-term outcomes, in terms of cure rate and complications, to the traditional costly meshes. Larger multicentre studies should confirm our results.

## Background

Stress urinary incontinence (SUI) is a prevalent disorder that is characterized by leakage of urine after physical stress [1]. Surgical procedures are widely performed for women with failed conservative management or women seeking definitive treatment [2]. Mid-urethral sling (MUS) is a highly effective, minimally-invasive, surgical approach for SUI that is based on the passing of a small band of a synthetic mesh into either the retropubic space (known as tension-free vaginal tape, TVT) or through the obturator foramen (known as trans-obturator tape, TOT or TVT-O, through the inside-out route) [3]. Previous reports showed that MUS achieves a cure rate of up to 94% [4], with a low risk of postoperative complications, such as visceral injuries and retention [5]. The current body of evidence demonstrated a well-established short- and long-term efficacy profile of MUS [6–8].

Nonetheless, TOT is a costly procedure, especially in developing countries, due to the need for prefabricated slings and specific needles or passers to reduce the risk of injuries to surrounding structures [9]. Thus, a number of surgeon-tailored meshes were proposed to reduce the cost of the procedure, with maintaining the postoperative and long-term outcomes [10]. Previously, we presented the short-term outcomes of surgeon-tailored mesh in patients with SUI undergoing TOT [11]. In this report, we aim to highlight the two-year outcomes of surgeon-tailored mesh in terms of subjective and objective cure rates, as well as late complications.

## Materials and methods

The protocol of the current trial was approved by the local ethics committee of the Faculty of Medicine for Girls Al-Azhar University, Cairo, Egypt. All procedures run in compliance with the standards of the Declaration of Helsinki [12]. We prepared the following manuscript in concordance with CONSORT guidelines [13].

### Study design and patients

We performed a randomized, open-label comparative trial that recruited women with SUI who were scheduled to undergo TOT at Al Zahraa University Hospital through the period from the end of December 2016 to the end of April 2021. Women were considered eligible if they fulfilled the following criteria: female patients aged more than 18 years old, patients with an established diagnosis of genuine SUI, and patients who did not exhibit a significant response to conservative management. We included women regardless of their parity status. On the other hand, we excluded patients with mixed incontinence, urge incontinence, acute infection, malignancies, overactive bladder, bleeding disorders, internal sphincteric deficiency, concomitant prolapse, post-void residual urine more than 150 cc, and/or bladder capacity less than 250 cc. Pregnant and lactating women were excluded as well.

Eligible patients were randomly allocated in a 1:1 ratio to receive traditional TOT mesh or surgeon-tailored polyethylene mesh by a computer software program (www.Randmizer.org), and allocation sequences were done by opaque closed envelopes.

### Study’s procedures and follow-up

All eligible women were subjected to standardized preoperative history taking and local gynecological examination. Patients underwent routine abdominopelvic ultrasonography (US) and cystometry by Andromeda Urodynamic apparatus.

The full surgical procedure was described previously [11]. After placing the patients in the lithotomy position, the surgeon introduced a 16 French catheter into the bladder and inflated the balloon. Then, the vaginal wall was incised in the midline for a one-cm distance, approximately one cm from the urethral meatus. This was followed by the incision of mucosa and submucosa, and dissection of periurethral fascia bilaterally towards the inferior pubic ramus. A skin incision was then performed medial to the genito-femoral fold at the base of the adductor longus tendon, approximately at the level of the clitoris. A needle with fixed tape was introduced from the obturator to the vaginal incisions. Patients in the surgeon-tailored polyethylene mesh group received 30 × 1 cm polypropylene strips, which were prepared by splitting the 30 × 30 prolene mesh and re-sterilized by plasma machine. All patients were asked to cough to assess continence after the bladder was filled with 250 ml saline. The skin and vaginal incisions were closed by 3-0 vicryl in sub-cuticular and interrupted fashions, respectively. All patients were followed up for two years.

### Study's outcomes

The primary outcome of the present study was the two-year objective success rate. The objective success year was categorized into the following three categories: cure, which donates that the women have no stress-induced urine leak on cough test and that the women exhibited satisfactory control of their urine; improved, which donates that the women had urine leak on severe exertion only and they express subjective improvement in their symptoms; and failure, which donates that the women still exhibit the same level of preoperative urine leak on cough test and they are not satisfied with their condition. The secondary outcomes of this study included the incidence of late postoperative complications, such as reoperation, mesh erosion, de novo urgency, and dyspareunia.

### Statistical analysis

Retrieved data were summarized and processed with IBM SPSS statistical software (version 25). Frequencies were used to describe gynecological history, Ultrasound findings, postoperative complications, and success rates. On the other hand, age, ALPP, duration surgery, and hospital stay were summarized, according to normality, into mean (± standard deviation [SD]) or median (range). The hypothesize of significant differences between the type of mesh and primary or secondary outcomes was challenged using the independent t-test or Chi-square test for continuous and categorical data, respectively. *P* value < 0.05 was regarded as statistically significant.

## Results

**Fig. 1:**
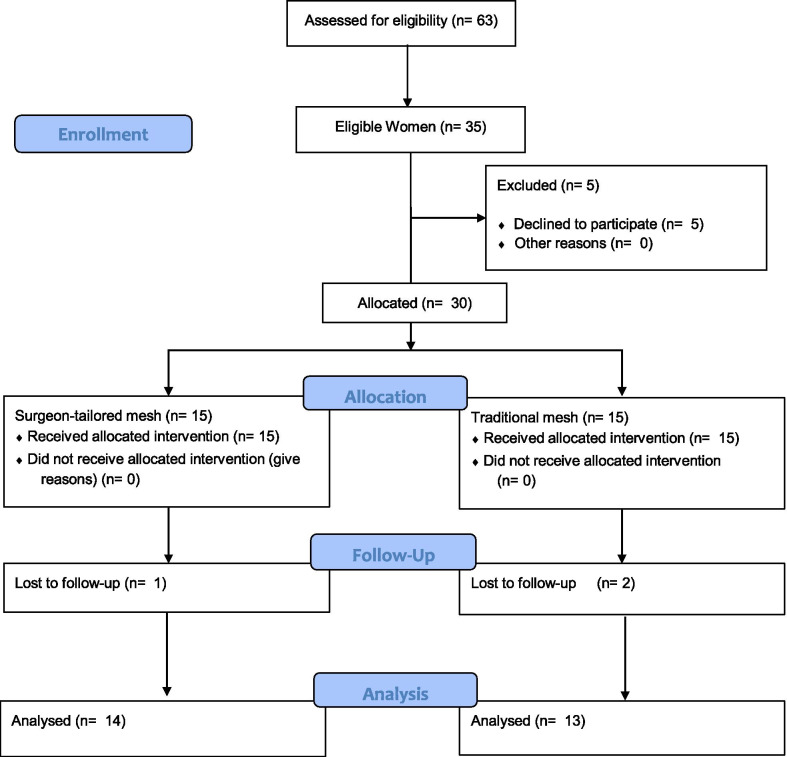
Checklist of the study

Initially a total of 63 women were screened for eligibility; of them, 35 women were deemed eligible and 30 of them agreed to sign the informed consent. The 30 women were randomly allocated to the traditional TOT mesh (n = 15 women) or surgeon-tailored polyethylene mesh (n = 15 women). At the end of follow-up, there were 13 women in the traditional TOT mesh group and 14 patients in the surgeon-tailored polyethylene mesh group (Fig. 1).

The mean age between the traditional TOT mesh and the surgeon-tailored polyethylene mesh groups was comparable (46.79 ± 7.567 vs. 40.67 ± 7.93 years old; *p* = 0.85). There were no significant differences between both groups in terms of parity (*p* = 0.36), number of previous vaginal deliveries (*p* = 0.13), number of previous Cesarean section (*p* = 0.18), use of pad (*p* = 0.63), and residual urine by US (*p* = 0.39). The mean operative time was slightly lower in the surgeon-tailored mesh group (15.67 ± 2.06 vs. 20.86 ± 3.35 min in the traditional TOT mesh group), without statistically significant difference (*p* = 0.075). Likewise, the mean ALPP was comparable between both groups (25.72 ± 6.87 vs. 33.72 ± 9.73, respectively; *p* = 0.51). The incidence of intraoperative vaginal wall injury was 0% in the surgeon-tailored polyethylene mesh group, compared to 7.7% in the traditional TOT mesh group (*p* = 0.34). The incidence of UTI or vaginal infection (23.1% versus 0% in the traditional TOT mesh and the surgeon-tailored polyethylene mesh groups, respectively), with *p* = 0.088 (Table 1).

**Table 1:** Perioperative Characteristics of the Follow-up Cohort (n = 27)

Variables	Traditional mesh (n = 13)	Surgeon-tailored mesh (n = 14)	*P* value
Age in years, mean ± SD	46.79 ± 7.567	40.67 ± 7.93	0.85
Parity, no. (%)			
P2	2 (15.4%)	5 (28.6%)	0.36
P3	7 (53.8%)	4 (28.4%)	
P4	2 (15.4%)	0	
> P4	2 (15.4%)	5 (28.6%)	
MOD, no. (%)			0.13
NVD	7 (53.8%)	5 (28.6%)	
CS	6 (46.2%)	9 (71.4%)	
Use of pad, no. (%)	2 (15.4%)	1 (7.1%)	0.63
Residual urine by US, no. (%)	2 (15.4%)	0	0.39
Operative time in minutes, mean ± SD	15.67 ± 2.06	20.86 ± 3.35	0.075
Intraoperative vaginal wall injury, No. (%)	1 (7.7%)	0	0.34
ALPP, mean ± SD	25.72 ± 6.87	33.72 ± 9.73	0.51
UTI or vaginal infection, No. (%)	3 (23.1%)	0	0.088

**Fig. 2:**
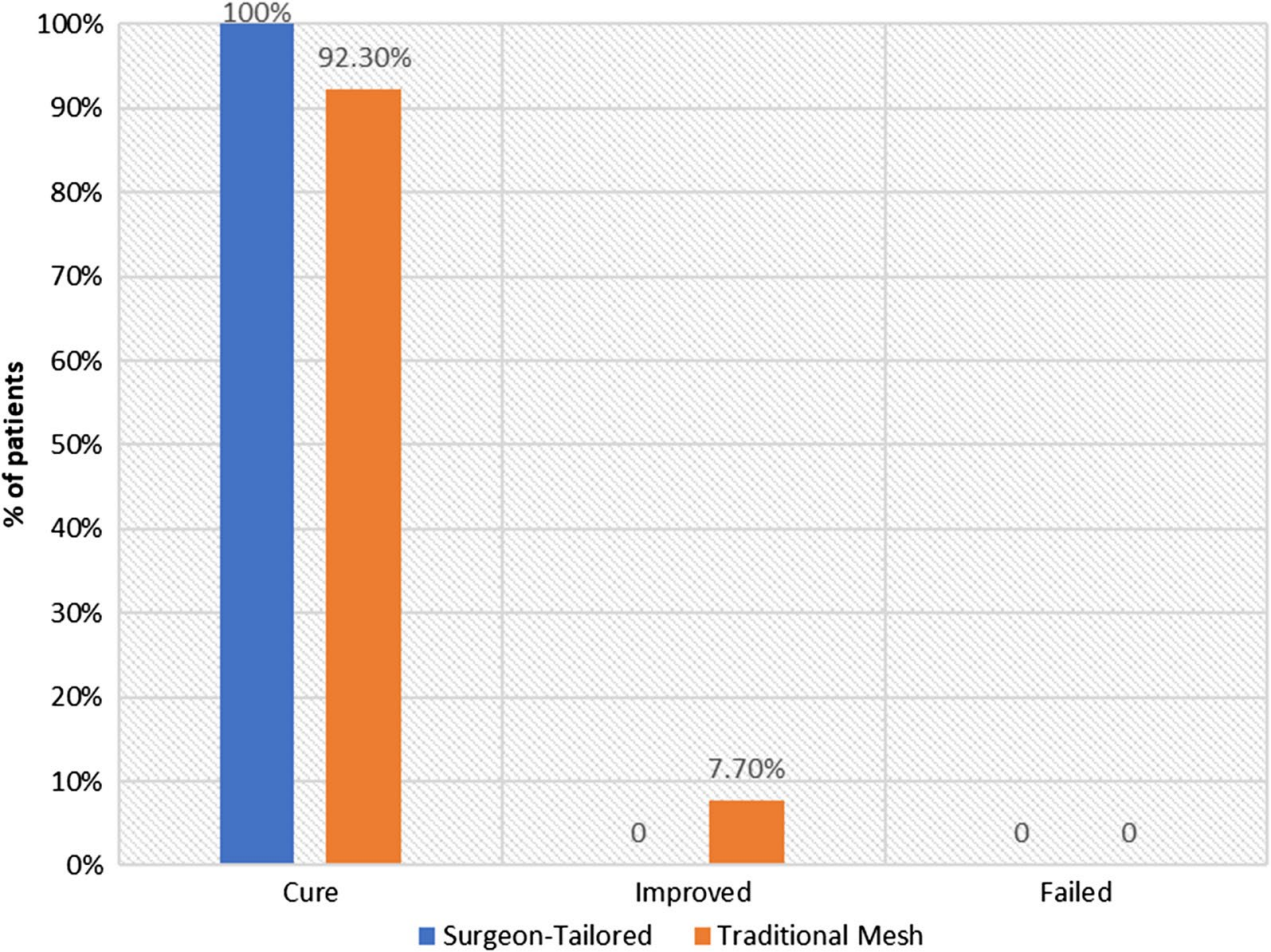
Outcome of the procedure

Concerning the primary outcome of the present study, the cure rate was 100% in the surgeon-tailored polyethylene mesh (n = 14) and 92.3% in the traditional TOT mesh group (*p* = 0.39). One woman reported improved symptoms in the traditional TOT mesh group. There were no reported failures in both groups (Fig. 2). With regard to safety, the incidence of de novo urgency was 0% in the surgeon-tailored polyethylene mesh group, compared to 7.7% in the traditional TOT mesh group (*p* = 0.34). None of the women in both groups reported mesh erosions, dyspareunia, or need for reoperation (Table 2).

**Table 2:** Safety outcomes of the follow-up cohort (n = 27)

Variables	Traditional mesh (n = 13)	Surgeon-tailored mesh (n = 14)	*P* value
De novo urgency, no. (%)	1 (7.7%)	0	0.34
Mesh erosions, no. (%)	0	0	–
Dyspareunia, no. (%)	0	0	–
Need for reoperation, no. (%)	0	0	–

## Discussion

Although there is a plethora of evidence about the efficacy and safety of surgical modalities for SUI, the published literature still lacks high-quality evidence about the long-term outcomes of new approaches aiming to improve the financial outcomes of these modalities [14, 15]. Thus, we conducted the present trial to highlight the two-year outcomes of surgeon tailored mesh in terms of subjective and objective cure rates and late complications. Our results showed the lack of statistically significant difference between surgeon-tailored mesh and traditional mesh in terms of cure rate (100% vs. 85.7%, respectively). Both techniques had comparable de novo urgency, mesh erosion, and dyspareunia rates at the end of the second year of follow-up.

TOT is a widely utilized, minimally-invasive technique that has proven its usefulness in patients with SUI; the technique is associated with optimal cure rates and diminished risks of major postoperative complications, such as injuries to surrounding structures and retention [3, 5]. Previous reports showed that the TOT exhibited acceptable levels of cure rate and safety profile on long-term follow-up among women with SUI [8, 16]. TOT was proven to be associated with lower direct cost than laparoscopic Burch colposuspension [17]. However, it is well-recognized that the TOT procedure is associated with a considerable financial burden on the patients and healthcare systems due to the high cost of its equipment, including meshes and passengers. Mesh erosion can put a further cost burden on the patients [18]. Thus, the need for alternative, less costly alternatives were deemed critical, particularly in developing countries [19]. Previous reports showed that the use of tailored polypropylene mesh resulted in satisfactory results and a less costly procedure for patients with SUI. Our previous report confirmed such findings showing that the surgeon-tailored mesh was associated with a high cure rate similar to traditional mesh [11]. Nonetheless, the current body of evidence shows scarcity of the long-term outcomes of this modified cheap approach. The present report found that the surgeon-tailored was associated with high cure rates at the end of the second year of follow-up, which donates its satisfactory long-term outcomes. In concordance with our findings, ElSheemy et al. [9], showed that the use of tailored polypropylene mesh in women undergoing TVT was associated with a cure rate of 91%. Another long-term comparative study by the same group showed that the tailored polypropylene mesh was associated with a comparable cure rate to the traditional mesh in women undergoing TVT [10]. In a Chinese report, Chen et al. [18], reported that the tailored polypropylene mesh led to a cure rate of 96% at the end of the first year of follow-up. Our findings are in line with short and medium-term results reported by Imamura et al. [15], in which the TOT achieved higher cure rates than other procedures.

The surgical management of SUI can be associated with a high rate of treatment failure and reoperation. It was previously noted that up to 4% of patients undergoing surgery need reoperation due to recurrent SUI. Nonetheless, the rate of reoperation is not consistent across various surgical approaches for SUI [20]. TOT is thought to increase the risk of reoperation due to synthetic mesh, which may be exposed in the long term and need removal. The synthetic mesh may also lead to chronic pain and voiding dysfunction requiring reoperation [3]. Several risk factors were implicated in the need for reoperation among women undergoing TOT, such as obesity, diabetes, revision surgery, and mixed UI [21]. More recently, Illiano et al. [22], noted that technical errors play a significant role in TOT failure and reoperation, which can be reduced by translabial ultrasound. However, it should be noted that long-term studies demonstrated a low rate of TOT failure and need for reoperation, with a follow-up period of nearly 13 years [23]. Other systematic reviews demonstrated similar findings [8, 16]. In the present study, we demonstrated that the use of surgeon-tailored mesh was associated with a similar risk of failure and reoperation compared to traditional mesh. Our findings are similar to the reports by ElSheemy et al. [9, 10] and Chen et al. [18].

To our knowledge, only a few reports have attempted to explore the outcomes of surgeon-tailored mesh for SUI patients undergoing TOT; nonetheless, we acknowledge the existence of several limitations in our trial. Our study was limited to single-center only, and all procedures were done by the same surgeons, which can affect the generalizability of our findings. In addition, the sample size was relatively small. The postoperative voiding functions have not also been assessed using validated tools.

*In conclusion,* surgeon-tailored mesh for SUI patients undergoing TOT is a cost-effective strategy that is associated with a comparable long-term cure rate and incidence of long-term complications to the traditional costly meshes. The present trial confirmed that the surgeon-tailored mesh was associated with a similar cure rate and incidence of late complications compared to the traditional meshes at the end of the second year of follow-up. Thus, surgeons should consider these low-cost meshes, especially in low-resource settings. Nonetheless, the current published literature is still limited by the low number of published cases and low quality of published reports; thus, further studies are needed to characterize the outcomes of surgeon-tailored mesh for SUI patients undergoing TOT. Larger multicenter studies should confirm our results.

## Data Availability

All relevant data are within the manuscript. Additional patient data can be requested from the corresponding authors upon reasonable reason.

## Abbreviations

TOT: Trans obturator tape
SUI: Stress urinary incontinence
MUS: Mid-urethral sling
PVS: Pubo-vaginal sling
TVT: Tension-free vaginal tape
ALPP: Abdominal leak point pressure
US: Ultrasonography
